# In in vivo evaluation of the anti-inflammatory and analgesic activities of compound Muniziqi granule in experimental animal models

**DOI:** 10.1186/s12906-016-0999-y

**Published:** 2016-01-22

**Authors:** Juanjuan Cheng, Tingyun Ma, Wei Liu, Hanxue Wang, Jizong Jiang, Yue Wei, Hemiao Tian, Nan Zou, Yudan Zhu, Hailian Shi, Xuemei Cheng, Changhong Wang

**Affiliations:** 1 Institute of Chinese Materia Medica, The MOE Key Laboratory for Standardization of Chinese Medicines and The SATCM Key Laboratory for New Resources and Quality Evaluation of Chinese Medicines, Shanghai University of Traditional Chinese Medicine, 1200 Cailun Road, Shanghai,, 201203 China; 2 Shanghai R&D Center for Standardization of Chinese Medicines, 199 Guoshoujing Road, Shanghai,, 201210 China

**Keywords:** Anti-inflammation, Analgesic, Compound Muniziqi granules, HPLC

## Abstract

**Background:**

Compound Muniziqi granule (MNZQ), a traditional Uighur medicinal preparation, comprises 13 species of medicinal plants. MNZQ is traditionally used for regulating body immunity, modulating inflammation and pain, detoxification, and inhibiting tumor growth. This study aims to scientifically evaluate the anti-inflammatory and analgesic activities of MNZQ, support its clinical use and further research with scientific evidence.

**Methods:**

The analgesic activity of MNZQ was evaluated using hot plate test and acetic acid-induced abdominal writhing test. Acute inflammation was evaluated using xylene-induced ear edema and carrageenan-induced paw edema models, while chronic inflammation was evaluated using cotton pellet-induced granuloma model.

**Results:**

MNZQ exerted analgesic activities with a significant dose-dependent increase in latency in the hot plate test. The percentage inhibition suggested that MNZQ exhibited analgesic activities in the central nervous system. Meanwhile, MNZQ at 0.8, 2.4, and 7.2 g/kg strongly inhibited the acetic acid-induced writhing response by 25.22 % (*p* < 0.01), 44.60 % (*p* < 0.001), and 49.41 % (*p* < 0.001), respectively. MNZQ also exerted analgesic activities in the peripheral nervous system. Moreover, MNZQ was demonstrated a significant anti-inflammatory effect against xylene-induced edema in a dose-dependent manner. The percentage inhibition was 22.24 % (*p* < 0.01) at the highest dosage of 7.2 g/kg. MNZQ at 1.62 and 4.86 g/kg significantly reduced carrageenan-induced rat hind paw edema by 82.43 % and 84.32 % (*p* < 0.001), respectively, 1 h after injecting carrageenan, and the inhibitory effect lasted for 5 h. MNZQ also exerted a significant anti-inflammatory effect against cotton pellet-induced granuloma formation. MNZQ at 1.62 and 4.86 g/kg could inhibit granuloma formation by 17.07 % and 17.60 %, respectively, whereas the percentage inhibition of diclofenac was 33.12 %.

**Conclusions:**

The results obtained suggest that MNZQ possesses potential anti-inflammatory and analgesic activities. This study provides a scientific basis for the use of MNZQ in alleviating pain and treating inflammatory disorders.

## Background

Inflammation has become the major focus of global scientific research in all human and animal diseases [1]. Many physical, chemical, and biological stimuli or some of their combinations can cause nonspecific inflammation in injured tissues. Bacterial and viral infections can cause many signaling molecules, macrophages, monocytes, and neutrophils to produce inflammatory mediators such as nitric oxide, prostaglandin, and tumor necrosis factor (TNF-α) [2]. Nonsteroidal anti-inflammatory drugs (NSAIDs) are widely used inflammation treatments worldwide [3, 4]. However, the applications of NSAIDs for gastric ulcer, kidney damage, bronchitis, and cardiovascular diseases are limited by their side effects [5]. Therefore, developing anti-inflammatory drugs from traditional medicines with few side effects is imperative [6].

Compound Muniziqi granule (MNZQ), a popular traditional Uighur medicine (TUM), is recorded in the Ministry of Health of the People’s Republic of China Pharmaceutical Standards–Uighur Medicine [7]. As described in Table 1, MNZQ comprises 13 species of medicinal plants, including seeds of *Peganum harmala*, *Cichorium intybus*, *Dracocephalum moldavica*, *Ocimum basilicum*, *Althaea rosea*, and *Nigella glandulifera*; fruits of *Pimpinella anisum*; roots of *Apium graveolens*, *Glycyrrhiza uralensis* and *Cichorium intybus*; Cortex of *Foeniculum vulgare*; and herbs of *Matricaria chamomilla* and *Cymbopogon caesius*. According to TUM, excess or deficiency in one or more of the four body fluids, namely, Khan (blood), phlegm, Sapra (yellow bile), and Savda (black bile), can influence human metabolism and cause diseases [8, 9]. As a maturative agent of the four abnormal body fluids, MNZQ can help these fluids recover from imbalances and treat diseases. In clinical settings, MNZQ can effectively regulate hormone synthesis and metabolism, and treat endocrine disorder-induced acne, chloasma, dysmenorrhea, menopausal syndrome, and inflammation of the skin or female genitalia [10].

**Table 1:** Information of components in Compound Muniziqi granules (MNZQ)

Botanical name	Herbal name	Part used	Chinese name	Ratio	Voucher no
*Peganum harmala* L.	Pegani semen	Seed	Luo-Tuo-Peng-Zi	2	Y120514
*Pimpinella anisum* L.	Pimpinellae anisi fructus	Fruit	Hui-Qin-Guo	2	Y130142
*Foeniculum vulgare Mill.*	Foeniculi cortex	Root bark	Hui-Xiang-Gen-Pi	2	Y130408
*Dracocephalum moldavica* L.	Dracocephali semen	Seed	Xiang-Qing-Lan-Zi	1	Y120923
*Ocimum basilicum* L.	Ocimi basilici semen	Seed	Luo-Le-Zi	1	Y110836
*Althaea rosea* (L.) Gavan.	Althaea semen	Seed	Shu-Kui-Zi	1	Y130303
*Nigella glandulifera* Freyn et Sint.	Nigellae semen	Seed	Hei-Zhong-Cao-Zi	1	Y120119
*Apium graveolens* L.	Apii radix	Root	Qin-Cai-Gen	1	Y130312
*Cichorium intybus* L.	Cichorii radix	Root	Ju-Ju-Gen	1	Y130212
*Cichorium intybus* L.	Cichorii semen	Seed	Ju-Ju-Zi	1	Y120812
*Glycyrrhiza uralensis* Fisch.	Glycyrrhizae radix Et Rgizoma	Root	Gan-Cao	1	Y130141
*Matricaria chamomilla* L.	Chamomillae herba	Herba	Yang-Gan-Ju	1	Y120927
*Cymbopogon caesius* (Ness) Stapf.	Cymboponis herba	Herba	Xiang-Mao	1	Y121112

Most of constituents of medicinal plants in MNZQ have potential anti-inflammatory activities. *P. harmala* exerts some pharmacological effects, including antibacterial, anti-inflammatory, analgesic, antipruritic, parasite-resistant, and antirheumatic effects [11]. For centuries, *M. chamomilla* and *Glycyrrhiza* have been widely used to eliminate inflammation and to treat various inflammatory disorders such as eczema, ulcers, gout, neuralgia, and rheumatic pains [12]. *Glycyrrhiza* species have also been used worldwide to treat injury or swelling. Meanwhile, *D. moldavica*, *N. glandulifera*, *C. intybus*, and *O. basilicum* exert anti-inflammatory effects [13]. However, it is yet to be determined whether MNZQ maintains strong anti-inflammatory and analgesic activities. Laboratory-based ethnopharmacological investigations of MNZQ in a broad context may impart complete understanding of TUM practice and use in China.

In recent years, few studies have shown that MNZQ can strongly inhibit dinitrofluorobenzene-induced allergic contact dermatitis and chloasma [14, 15]. MNZQ treats various pelvic inflammatory diseases possibly by regulating the expression of TNF-α, IL-1β, and IL-10 [16]. Tamoxifen citrate tablets combined with MNZQ alleviate the pain caused by mammary gland hyperplasia [17]. However, there is insufficient data to support the anti-inflammatory and analgesic activities of MNZQ. The aim of this study is to provide scientific evidence for the ethnopharmacological use of MNZQ in alleviating pain and treating inflammatory disorders.

## Methods

### Reagents and materials

MNZQ was provided by Xinjiang Uighur Pharmaceutical Co., Ltd. (Xinjiang, China; Batch No. 1212522). HPLC grade acetonitrile was purchased from Fisher Scientific (Fair Lawn, NJ, USA). Ultrapure water was obtained from a Milli-Q water purification system (Billipore, MA, USA). Acetic salicylic acid (ASA) was purchased from Huayin City Jinqiancheng Pharmaceutical Co., Ltd. (Shanxi, China; Batch No. A1111002). Diclofenac sodium and carrageenan were purchased from Sigma-Aldrich Co. (St. Louis, MO, USA). Xylene and acetic acid were purchased from Sinopharm Chemical Reagent Co., Ltd. (Shanghai, China). The standard reference compounds of chlorogenic acid, caffeic acid, ferulic acid, liquiritin, harmaline, harmine, apigenin 7-O-glucoside, and isoliquiritin were purchased from Shanghai R&D Center for Standardization of Chinese Medicines. All other chemicals used were of analytical grade.

### Experimental animals

All experimental animals, including male and female Kunming (KM) mice weighing 20–25 g and male adult Wistar rats weighing 180–200 g (Certificate No. SYXK, Shanghai, 2009–0069), were obtained from SLAC Laboratory Animal Co., Ltd. (Shanghai, China) and housed by the Animal Center of Shanghai University of Traditional Chinese Medicine. The rats were housed in an air-conditioned room with a temperature of 22–24 °C and a relative humidity of 60 %–65 % with a 12 h dark–light cycle (light on from 7:00 to 19:00). All animals were provided with standard pellet diet and water spontaneously. The animals were acclimatized to the facilities for 7 days and then allowed to fast with free access to water 12 h before the experiments. The animal studies were conducted in accordance with the Institute’s Guide for the Care and Use of Laboratory Animals and were approved by the Ethical Committee of Shanghai University of Traditional Chinese Medicine (Approval No. ACSHU-2014-200, approved in 16 July, 2014).

## High performance liquid chromatography (HPLC) fingerprinting and quantitative determination

### Instruments and chromatographic conditions

An HPLC system equipped with an Agilent 1260 series (Agilent technologies, Waldbronn, Germany) LC system was used. This LC System constituted a G1311B quaternary pump, a G1321B degasser, a G1367E automatic sampler, a G1316A thermostated column compartment, and G1315C diode array detection. All samples were separated on a C_18_ chromatographic column (4.6 mm × 250 mm, 5 μm, Boston Lunna Clone, Boston Analytics, Inc., USA) with a gradient system of acetonitrile (A) and ammonium acetate buffer (B). The elution program is described in Table 2. The flow rate was 1 mL/min, the injection volume was 20 μL, and the column temperature was maintained at 30 °C. The detection wavelength was set at 335 nm. Ammonium acetate buffer was prepared by dissolving 3.4 g of ammonium acetate in 500 mL of pure water, and glacial acetic acid (3 mL) was added to adjust pH to 4.5.

**Table 2:** Gradient elution program for HPLC

Time (min)	Flow (mL/min)	% acetonitrile (A)	% ammonium acetate buffer, PH 4.5 (B)
0~5	1	5	95
5~25	1	18	82
25~30	1	18	82
30~45	1	32	68
45~60	1	70	30

### Standard preparation

The standard stock solutions of chlorogenic acid (0.52 mg/mL), caffeic acid (0.54 mg/mL), ferulic acid (0.54 mg/mL), liquiritin (0.52 mg/mL), harmaline (1.04 mg/mL), harmine (0.82 mg/mL), apigenin 7-O-glucoside (0.48 mg/mL), and isoliquiritin (0.50 mg/mL) were prepared in methanol and stored at 4 °C. Working solutions of these standard solutions were prepared by dilution of the stock solution.

### Sample preparation

A 15 g aliquot of MNZQ sample (Batch No. 1210481) was completely dissolved in 20 mL of 0.05 M hydrochloric acid solution and extracted three times with 40 mL of ethyl acetate in a separatory funnel. The organic supernatants were combined to evaporate to dryness. The residue was dissolved in 2 mL of methanol, and then the solution was filtered through a Millipore filter (0.45 μm) to obtain the sample solution before injection into LC system for analysis.

### Fingerprinting and quantitative determination

Working solutions of standard solutions and sample solution were injected into LC system for analysis fingerprinting of MNZQ, and the individual peak in fingerprint of MNZQ was confirmed by comparing with reference standards. Further the content of each characteristic peak was quantitatively determined by a validated method (no published data).

### Drug administration

MNZQ was dissolved in distilled water and administered orally. The MNZQ dosage for animal was extrapolated from the human daily dose of 18 g based on body weight. On the basis of the equivalent dose rate conversion for animal and human body surface areas, the middle dosages for mice and rats were 2.4 and 1.62 g/kg, respectively. For three times the gradient of the middle dose, the low dosages for mice and rats were 0.8 and 0.54 g/kg, and the high dosages were 7.2 and 4.86 g/kg, respectively. The dose volume was 20 mL/kg for mice and 10 mL/kg for rats, respectively. During the period of administration, rats were kept under regular observation for any adverse effect, including mortality. Other behaviors such as body weight, food and water intake, urination, locomotor activity, hair luster, etc., were also observed over a period of seven days.

## Analgesic activity

Female and male mice were used for the hot plate test and acetic acid-induced writhing test to evaluate the analgesic activities of MNZQ in the central and peripheral nervous systems, respectively.

### Hot plate test

The hot plate test was performed as previously described [18, 19]. Adult female KM mice (20–25 g) were selected for this study. The mice were placed on the heated plate before the experiment. The temperature of the hot plate was maintained at 55 ± 0.5 °C. The latency between the placement and shaking or the licking of the hind paws or the jumping response of the animals was recorded as the latent response. The mice that exhibited latencies within 5–30 s were selected for the experiment. The selected mice were divided randomly into five groups of eight animals each. The pre-treatment reaction time of each mouse was recorded. The control vehicle (distilled water, 20 mL/kg, p.o.), ASA positive control (100 mg/kg, p.o.), and three doses of MNZQ (0.8, 2.4, and 7.2 g/kg, p.o.) were administered orally for six consecutive days. On day 7 at 1 h after oral administration, the post-treatment reaction time of each animal was recorded after 30, 60, 90, and 120 min [20]. If the pain domain values exceeded 60 s, then the cut-off time was set to 60 s to avoid scalding of mouse foot. The percentage inhibition was calculated by using the following formula: %Inhibition = [(Post-treatment Latency) − (Pre-treatment Latency)]/Pre-treatment Latency × 100.

### Acetic acid-induced abdominal writhing in mice

The test for the writhing study was performed as previously described [21]. The mice were randomly divided into the following five treatment groups of eight animals each: vehicle control (distilled water, 20 mL/kg, p.o.), ASA positive control (100 mg/kg, p.o.), and three doses of MNZQ (0.8, 2.4, and 7.2 g/kg, p.o.). These treatments were administered orally for six consecutive days. On day 7 at 1 h after oral administration, the mice were induced to writhing with 0.6 % acetic acid solution in normal saline injected intraperitoneally at a dose of 10 mL/kg. The frequencies of writhing (abdominal constrictions, pelvic rotation, and hind limb stretching) were recorded for 15 min, and the first time (recorded as latent period) writhing appeared after acetic acid injection was also recorded. The percentage analgesic activity was calculated using the following formula: %Inhibition = [Numbers of writhes (control) − Numbers of writhes (test)]/Numbers of writhes (control) × 100.

## Anti-inflammatory activity

### Xylene-induced ear edema in mice

The acute inflammatory activity of MNZQ was evaluated using xylene-induced ear edema in mice as previously described [22, 23]. The mice were randomly divided into the following five treatment groups of eight animals each: vehicle control (distilled water, 20 mL/kg, p.o.), ASA positive control (100 mg/kg, p.o.), and three doses of MNZQ (0.8, 2.4, and 7.2 g/kg, p.o.). These treatments were administered orally for six consecutive days. On day 7 at 1 h after oral administration, 30 μL of xylene was applied on both surfaces of right ear to induce edema. The left ear served as a control. The mice were sacrificed 30 min after xylene treatment by performing cervical dislocation. The right and left ears of the mice were removed with an 8-mm diameter cork borer, and the ears were weighed. The edema weight difference between the right and left ears of the same animal was measured. The percentage of inhibition compared with the control group was calculated using the following formula: %Inhibition = [(Weight of edema (control) − Weight of edema (test)]/Weight of edema (control) × 100.

### Carrageenan-induced rat hind paw edema

The test carrageenan-induced rat hind paw edema was evaluated as previously described [24]. The rats were randomly divided into the following five treatment groups of eight animals each: vehicle control (distilled water, 10 mL/kg, p.o.), diclofenac positive control (5 mg/kg, p.o.), and three doses of MNZQ (0.54, 1.62, and 4.86 g/kg, p.o.). These treatments were administered orally for six consecutive days. On day 7 at 1 h after oral administration, 0.1 mL of 1 % carrageenan in saline was injected into the plantar area of the rat right hind paw. The hind paw volumes before (0 h) the injection of carrageenan and 1, 2, 3, and 5 h after the injection of carrageenan were measured using a plethysmometer (Ugo Basile, Milan, Italy). The percentage of inhibition compared with the control group was calculated using the following formula: %Inhibition = [(Volume of edema (control) − Volume of edema (test)]/ Volume of edema (control) × 100.

### Cotton pellet-induced granuloma in rats

The chronic anti-inflammatory activity of MNZQ was evaluated using cotton pellet-induced granuloma in rats as previously described [25, 26]. The rats were randomly divided into the following five treatment groups of eight animals each. Each rat was anesthetized and shaven, and two sterilized cotton pellets weighing 20.00 ± 1.00 mg each were surgically implanted into both sides of the groin region of each rat on the first day. The next day after surgery, the rats underwent intragastric administration of distilled water, diclofenac, and MNZQ (grouping the same as that in the test of carrageenan-induced rat hind paw edema). After 7 d, all of the rats were sacrificed by performing cervical dislocation, and the pellets surrounded by granuloma tissue were carefully dissected. The pellets were cleared of surrounding tissues, weighed to obtain the moist weight. Then the moist pellets were dried overnight at 60 °C and weighed again to obtain the dry weight. The differences in moist or dry pellets weight between the test and control groups were calculated. The percentage of inhibition compared with the control group was calculated using the following formula: %Inhibition = [(Weight of pellet (control) − Weight of pellet (test)]/Weight of pellet (control) × 100.

### Statistical analysis

Experimental results were expressed as mean ± standard error (SEM) with eight animals in each group. Data were analyzed by one-way ANOVA between different test groups. SPSS 18.0 was used for all statistical analyses. Results were considered statistically significant at *p* < 0.05. All figures were generated using Graphpad Prism 5 Software.

## Results

### HPLC fingerprinting and quantitive determination

**Fig. 1:**
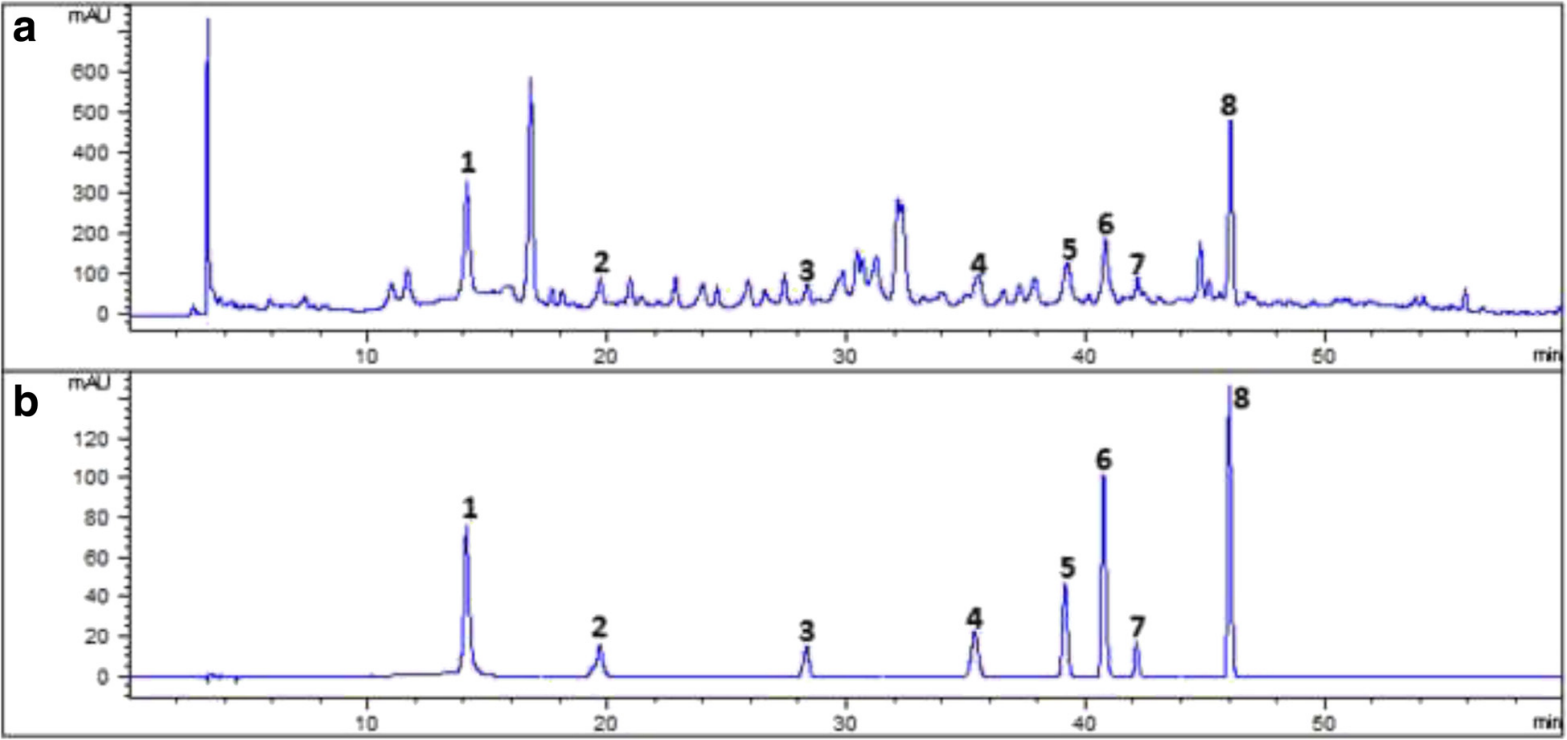
HPLC fingerprinting of MNZQ (**a**) and chromatogram of standard references (**b**) (1. chlorogenic acid, 2. caffeic acid, 3. ferulic acid, 4. liquiritin, 5. harmaline, 6. harmine, 7. apigenin 7-O-glucoside, and 8. isoliquiritin)

The results of HPLC fingerprinting of MNZQ were shown in Fig. 1. Eight characteristic peaks were identified as chlorogenic acid, caffeic acid, ferulic acid, liquiritin, harmaline, harmine, apigenin 7-O-glucoside, and isoliquiritin. Comparison of the HPLC chromatograms of individual herbal medicines with standard reference substances confirmed that liquiritin and isoliquiritin are from the characteristic compositions of *G. uralensis*, harmaline and harmine from *P. harmala*, chlorogenic acid from *P. anisum*, *M. chamomilla*, and *D. moldavica*, caffeic acid from *D. moldavica*, ferulic acid from *F. vulgare*, and apigenin 7-O-glucoside from *M. chamomilla* and *A. graveolens* roots. The contents of chlorogenic acid, caffeic acid, ferulic acid, liquiritin, harmaline, harmine, apigenin 7-O-glucoside, and isoliquiritin in MNZQ were determined as 17.29, 4.04, 3.73, 72.14, 270, 120, 2.77, and 10.48 μg/g, respectively.

## Analgesic activity

### Effects of hot plate

**Fig. 2:**
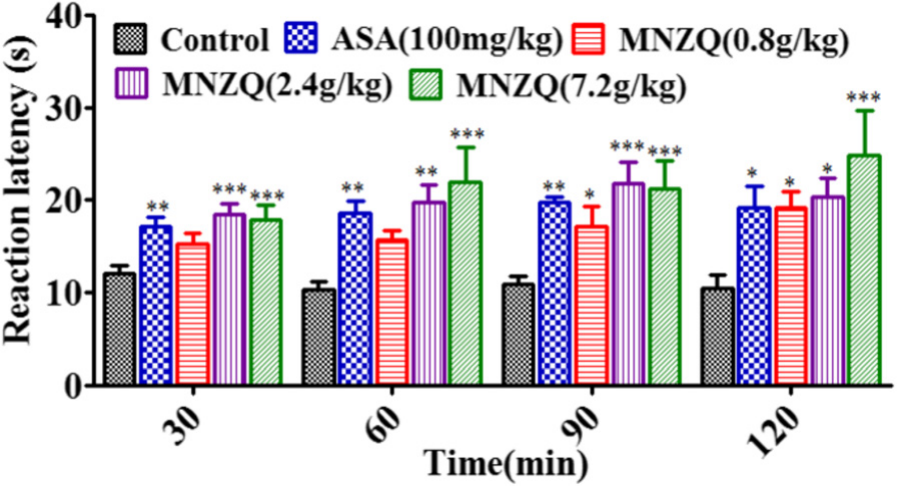
Effects of MNZQ on hot plate analgesic test in mice. The time between the placement of mouse and its shaking or licking of the hind paws on a hot plate for 30, 60, 90, and 120 min after administration was recorded as reaction time. Vehicle control mice were administered with distilled water, and ASA (100 mg/kg) was used as the positive control. Results are expressed as the mean ± SEM (*n* = 8) of the reaction time in seconds. *p < 0.05, **p < 0.01, ***p < 0.001 were considered significant when compared at the same time with the vehicle control (distilled water) after administration

The results of the hot plate test on the analgesic activities of MNZQ in the central nervous system were shown in Fig. 2. MNZQ at 2.4 and 7.2 g/kg showed significantly increased analgesic effect in the 30–120 min latency period after administration. The inhibition percentages of 2.4 and 7.2 g/kg MNZQ increased by 67.54 % and 64.64 % (*p* < 0.001) at 30 min after administration, and by 79.79 % (*p* < 0.01) and 101.47 % (*p* < 0.001) at 60 min after administration, respectively. Meanwhile, the inhibition percentages with positive control ASA (100 mg/kg) increased by 59.64 % and 73.20 % at 30 and 60 min after administration, respectively. However, MNZQ at 0.8 g/kg showed no significant analgesic activity at the 30 and 60 min latency periods after administration (*p* < 0.05). The inhibition percentages of 0.8, 2.4, and 7.2 g/kg MNZQ increased by 59.05 % (*p* < 0.05), 97.84 % (*p* < 0.001), and 95.13 % (*p* < 0.001) at 90 min after administration, and by 77.43 % (*p* < 0.05), 85.47 % (*p* < 0.05), and 112.38 % (*p* < 0.001) at 120 min after administration, respectively. Meanwhile, the inhibition percentages of the positive control ASA (100 mg/kg) increased by 83.78 % (*p* < 0.001) and 78.76 % (*p* < 0.05) at 90 and 120 min after administration, respectively. All differences were compared with the vehicle control (distilled water).

### Effects of acetic acid-induced abdominal writhing in mice

**Fig. 3:**
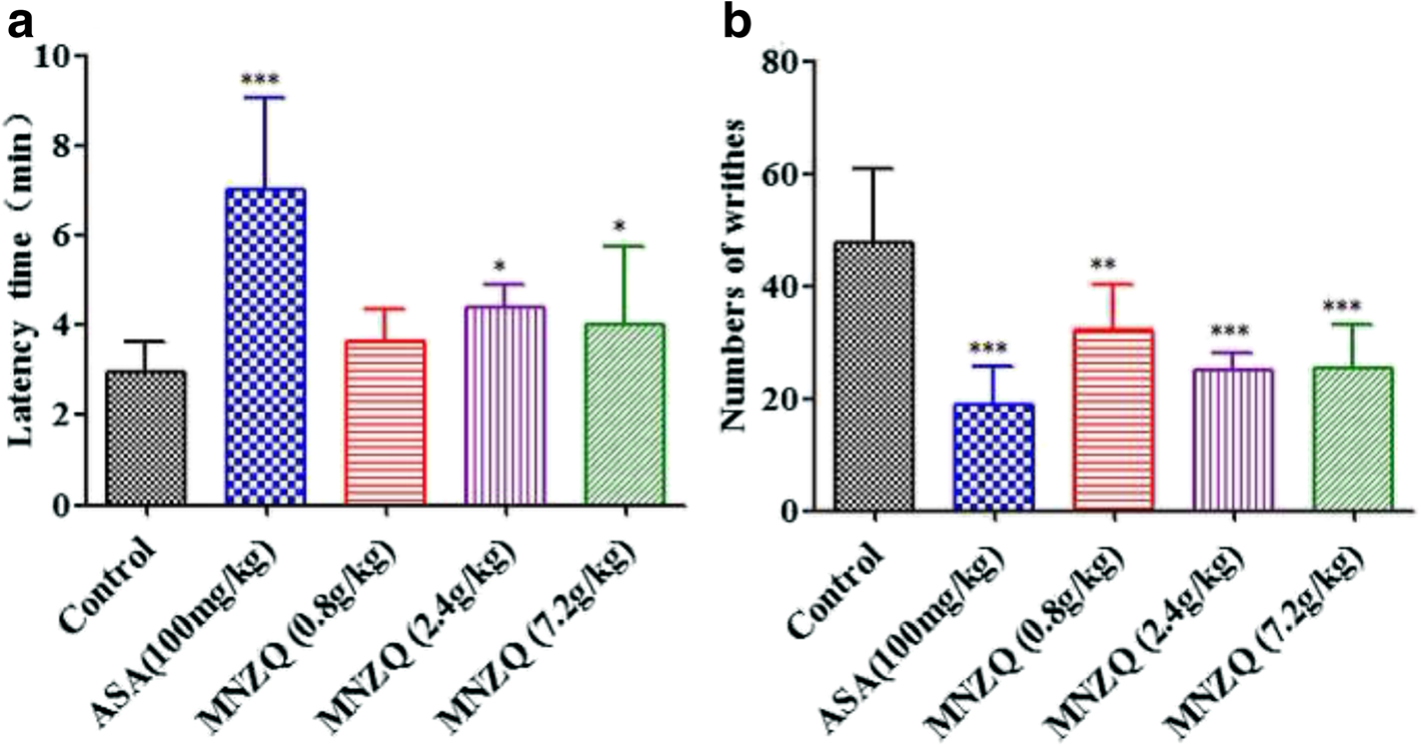
Effect of MNZQ on acetic acid-induced abdominal writhing in mice. Frequency of writhing within 15 min (**a**) and the latent period (**b**) after intraperitoneal injection of acetic acid. Vehicle control mice were administered with distilled water, and ASA (100 mg/kg) was used as the positive control. Results are expressed as the mean ± SEM (*n* = 8) of the reaction time in seconds. *p < 0.05, **p < 0.01, ***p < 0.001 were considered significant when compared with the vehicle control (distilled water) after administration

The peripheral analgesic activities of MNZQ on acetic acid-induced abdominal writhing in mice were shown in Fig. 3. MNZQ at 0.8, 2.4, and 7.2 g/kg significantly inhibited the acetic acid-induced writhing response within 15 min by 25.22 % (*p* < 0.01), 44.60 % (*p* < 0.001), and 49.41 % (*p* < 0.001), respectively. The positive control ASA (100 mg/kg) also strongly inhibited the writing response by 51.20 % (*p* < 0.001) (Fig. 3a). Compared with the control treatment, MNZQ at 2.4 and 7.2 g/kg significantly delayed the onset period of writhing behavior by 49.15 % and 51.88 % (*p* < 0.05), respectively. Meanwhile, the positive control ASA (100 mg/kg) delayed the latent period by 138.90 % (*p* < 0.001). However, 0.8 g/kg MNZQ did not significantly delay the latent period compared with the vehicle control (Fig. 3b).

## Anti-inflammatory activity

### Effects of xylene-induced ear edema in mice

**Fig. 4:**
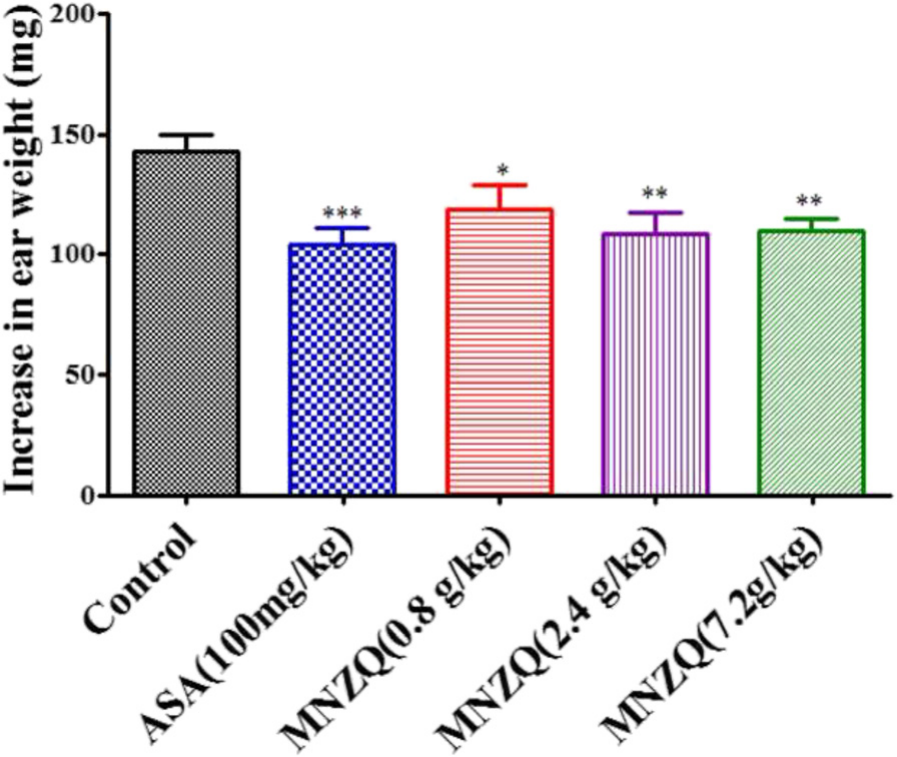
Effect of MNZQ on xylene-induced ear edema in mice. Mice were administered with MNZQ orally 1 h before xylene (30 μL/ear) was applied to the right ears of the mice. Vehicle control mice were administered with distilled water, and ASA (100 mg/kg) was used as the positive control. Results are expressed as the mean ± SEM (*n* = 8) of the percentage inhibition of edema. *p < 0.05, **p < 0.01, ***p < 0.001 were considered significant when compared with the vehicle control (distilled water) after administration

The dose dependent effects of MNZQ against acute inflammation in xylene-induce ear edema in mice were shown in Fig. 4. Compared with the control treatment, MNZQ at 0.8, 2.4, and 7.2 g/kg decreased ear edema by 17.18 % (*p* < 0.05), 22.17 % (*p* < 0.01), and 22.24 % (*p* < 0.01), respectively. The positive control ASA (100 mg/kg), meanwhile, inhibited ear edema by 25.80 % (*p* < 0.001).

### Effects of carrageenan-induced rat hind paw edema

**Fig. 5:**
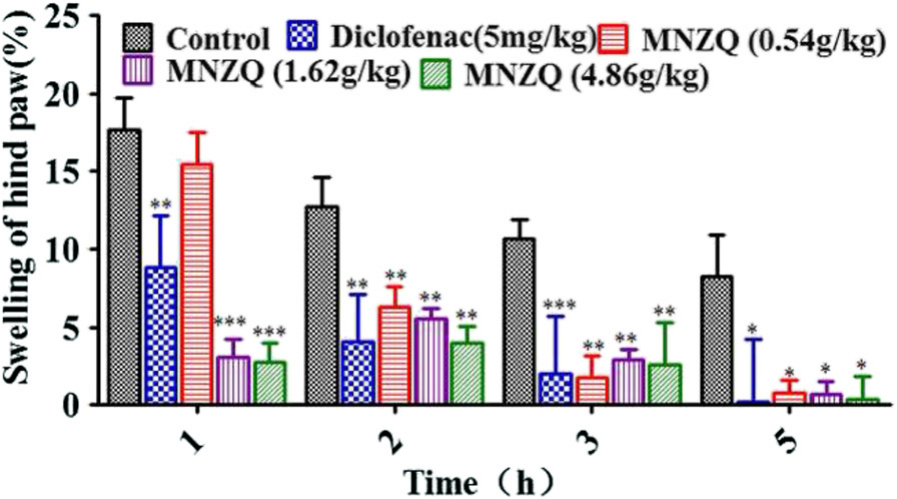
Effect of MNZQ on carrageenan-induced rat hind paw edema. Rats were administered orally with MNZQ 1 h before carrageenan injection to the right hind paw. Vehicle control mice were administered with distilled water, and diclofenac (5 mg/kg) was used as the positive control. Results are expressed as the mean ± SEM (*n* = 8) of the percentage inhibition of edema. *p < 0.05, **p < 0.01, ***p < 0.001 were considered significant when compared with the vehicle control (distilled water) after administration

The effects of MNZQ on acute inflammation in the carrageenan-induced rat hind paw edema were shown in Fig. 5. Compared with the control treatment, MNZQ at 1.62 and 4.86 g/kg significantly inhibited rat hind paw edema by 82.43 % and 84.32 % (*p* < 0.001), respectively, at 1 h after administration. MNZQ at 0.54 g/kg did not significantly inhibit edema. Low-dosage MNZQ took effect at 2 h after administration and prolonged to 5 h. Meanwhile, the positive control diclofenac (5 mg/kg) significantly decreased carrageenan-induced rat hind paw edema in a time-dependent manner (until 5 h after administration).

### Effects of cotton pellet-induced granuloma in rats

**Fig. 6:**
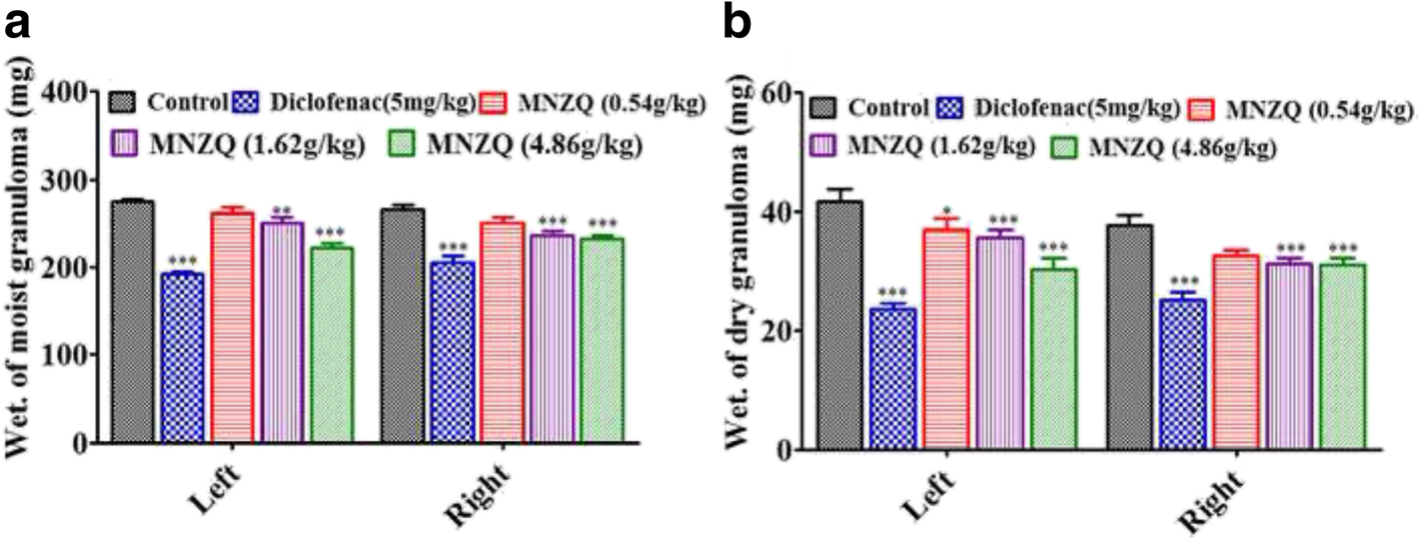
Effect of MNZQ on cotton pellet-induced granuloma in rats. Weight of moist granuloma (**a**) and dry granuloma (**b**). Vehicle control mice were administered with distilled water; diclofenac (5 mg/kg) was used as the positive control. Results are expressed as the mean ± SEM (*n* = 8) of the percentage inhibition of edema. *p < 0.05, **p < 0.01, ***p < 0.001 were considered significant when compared with the vehicle control (distilled water) after administration

The effects of MNZQ against chronic inflammation in cotton pellet-induced granuloma in rats were shown in Fig. 6. The moist weight of granuloma showed that MNZQ at 1.62 and 4.86 g/kg significantly inhibited left side granuloma formation by 8.81 % (*p* < 0.01) and 19.15 % (*p* < 0.001) and right side granuloma formation by 11.37 % (*p* < 0.001) and 12.84 % (*p* < 0.001), respectively, compared with the control treatment. In addition, the dry weight of granuloma reflected that MNZQ at 1.62 and 4.86 g/kg inhibited left side granuloma formation by 14.84 % (*p* < 0.05) and 27.40 % (*p* < 0.001) and right side granuloma by 17.07 % (*p* < 0.001) and 17.60 % (*p* < 0.001), respectively. Diclofenac (5 mg/kg), positive control, also significantly inhibited inflammation in both sides of cotton pellet (*p* < 0.001).

## Discussion

**Fig. 7:**
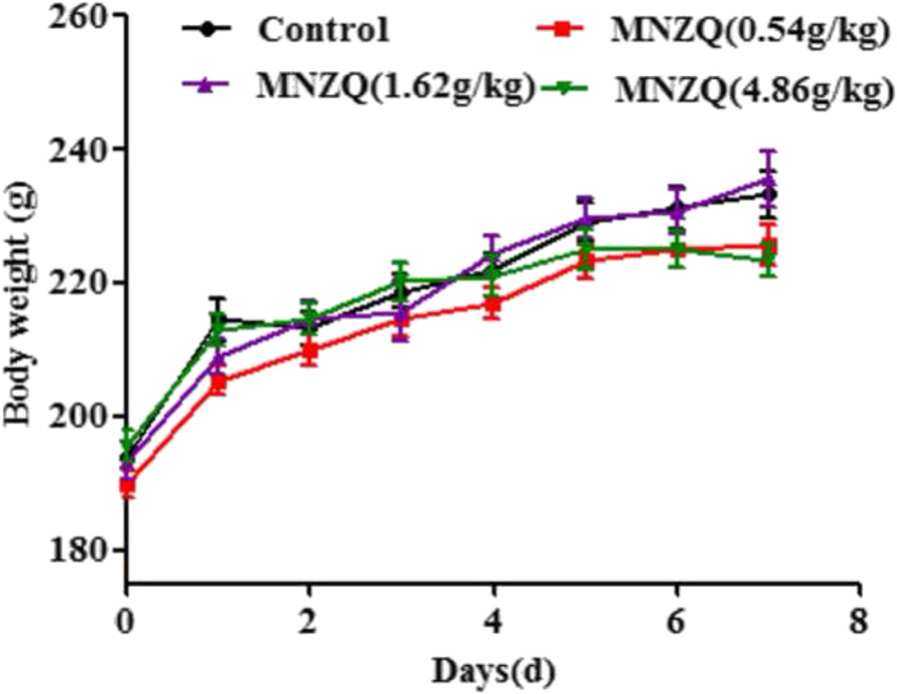
The body weight growth curves of rats after administration MNZQ. Vehicle control was administered with distilled water

During the period of drug administration for 7 days, food and water intake, urination, hair luster, locomotor activity, and behavior of animals in control and three drug treatment groups indicated no any significance. As shown in Fig. 7, the rats body weight growth curves of animals in control and three drug treatment groups shown similar trend without no significant difference (*p* > 0.05), as the experiment ongoing. These observations indicated that the high dose (4.86 g/kg for rats or 7.2 g/kg for mice) of MNZQ was safe.

Different models of thermal and chemical nociception in animals were used to evaluate the anti-inflammatory and analgesic activities of MNZQ. The hot plate test and acetic acid-induced abdominal writhing test were commonly used to assess central and peripheral activity models [27]. The two nociception models revealed the analgesic activities of MNZQ on the central and peripheral nervous systems. Among the composition of MNZQ, *P. harmala* seeds, *P. anisum*, and *D. moldavica* possibly contributed the greatest to the analgesic activities of the medicine. The seeds of *P. harmala* as one of the main medicinal plants in MNZQ, the main active components of *P. harmala* are beta-carboline alkaloids harmine and harmaline [28, 29]. It was reported that the alkaloid extract of *P. harmala* seeds, which mainly consists of harmine and harmaline, exerts anti-inflammatory and analgesic activities [30, 31]. It was reported that *P. anisum*, another main herbal component in MNZQ, can reduce morphine dependence and help relieve dysmenorrhea and the hot flash symptoms in menopausal women [32]. *D. moldavica* extracts also possess sedative and analgesic activities [33]. It is reported that the abdominal constriction is related to the sensitization of nociceptive receptors to prostaglandins [34, 35]. Local irritation produced by an intraperitoneal injection of acetic acid triggers the release pro-inflammatory cytokines such as IL-1, IL-6, IL-8 and TNF-α [36]. The hot plate test is assessed the participation of opioid, adenosine, and glutamate receptor systems [37]. These analgesic models suggested that the analgesic effect of MNZQ may be mediated by inhibiting the synthesis and release of prostaglandins and other pro-inflammatory cytokines such as IL-1, IL-6, IL-8, and TNF-α.

One chronic model and two acute models of inflammations were adopted in the present study to evaluate the anti-inflammatory effects of MNZQ. The model of xylene-induced ear edema in mice was linked to neurogenous edema release of inflammatory mediators such as histamine, kinin, and fibrinolysin [38, 39]. Carrageenan-induced rat hind paw edema model is widely used to evaluate anti-inflammatory activities [40]. Carageenan-induced edema is biphasic, the first phase (first 90 min) after carrageenan injection releases some chemical mediators of serotonin, histamine, bradykinin, and similar substances, whereas the second or late phase (after 90 min) is linked to the release of prostaglandins, proteases, and lysosomes [27, 41]. In addition, the late phase is related to the induction of cyclooxygenase in the hind paw [42]. Chronic inflammation was evaluated using a classic model of cotton pellet-induced granuloma in rats. The results of the present study revealed that MNZQ can significantly inhibit carrageenan-induced rat hind paw edema and effectively reduce cotton pellet granuloma.

Among the 13 species medicinal plants in MNZQ, *M. chamomilla* and *Glycyrrhiza* have been widely used to eliminate inflammation for centuries. Main beta-carboline alkaloids harmine can significantly decrease xylene-induced ear edema and carrageenan-induced rat hind paw edema [43]. It was confirmed that the mechanism on anti-inflammatory activity of *P. harmala* alkaloids is to inhibit myeloperoxidase [31]. There are increasing evidences that flavonoids and phenolic acids substance have good anti-inflammatory effects. The extract of *Glycyrrhiza* contains a large number of flavonoids, isoflavonoids, chalcones, and triterpene saponins, including liquiritin, isoliquiritin, glycyrrhizic acid, and liquiritigenin, which have been demonstrated potential anticancer, antiviral, and anti-inflammatory activities [44, 45]. Given its anti-inflammatory and analgesic properties, *M. chamomilla* has been used for centuries as a medicinal plant. Among the constituents of aqueous *M. chamomilla* extract, the main ingredients of apigenin 7-O-glucoside, apigenin exerts strong anti-inflammatory activity against pro-inflammatory agents. In rats, apigenin 7-O-glucoside can inhibit skin inflammation caused by the application of xanthine–oxidase and cumene hydroperoxide [45, 46]. Thus, these medicinal plants and their active components may be responsible for the anti-inflammatory activity of MNZQ.

The current study confirmed the anti-inflammatory and analgesic properties of MNZQ through several animal tests. However, further studies are warranted to elucidate the exact mechanism of action and confirm the chemical compounds responsible for the anti-inflammatory and analgesic effects of MNZQ.

## Conclusions

MNZQ exhibits significant anti-inflammatory activities and analgesic effects on the central and peripheral nervous systems. This study provides scientific foundation for the ethnobotanical uses of MZNQ in alleviating pain and treating inflammatory disorders.
